# Atrial Fibrillation as a Possible Adverse Reaction to Evolocumab

**DOI:** 10.7759/cureus.15467

**Published:** 2021-06-05

**Authors:** Ramy Abdelmaseih, Randa Abdelmasih, Mustajab Hasan, Alan Hamza

**Affiliations:** 1 Internal Medicine, University of Central Florida College of Medicine, Ocala, USA

**Keywords:** evolocumab, pcsk-9 inhibitor, hyperlipidemia, atrial fibrillation, arrhythmia, ekg

## Abstract

Evolocumab is a recently FDA-approved proprotein convertase subtilisin/kexin type 9 inhibitor (PCSK9i) that reduces the risk of myocardial infarction, stroke, and coronary revascularization in individuals with established atherosclerotic cardiovascular disease. We report an extremely rare possible side effect, atrial fibrillation (AF), encountered with evolocumab to increase the awareness among physicians of such a possibility.

## Introduction

Evolocumab, a proprotein convertase subtilisin/kexin type 9 inhibitor (PCSK9i) is a novel low-density lipoprotein (LDL) lowering agent that has been recently approved by the FDA to reduce the risk of myocardial infarction, stroke, and coronary revascularization in individuals with established atherosclerotic cardiovascular disease, alone or in combination with other lipid-lowering agents, and for treatment of patients with primary hyperlipidemia including familial hypercholesterolemia. The most common adverse effects reported in clinical trials were: nasopharyngitis (6-11%), upper respiratory tract infection (URTI) (8-9%), diabetes (9%), and injection-site reactions [1]. Here we report the first case, to our knowledge, of atrial fibrillation (AF) as a possible adverse reaction to evolocumab.

## Case presentation

**Figure 1:**
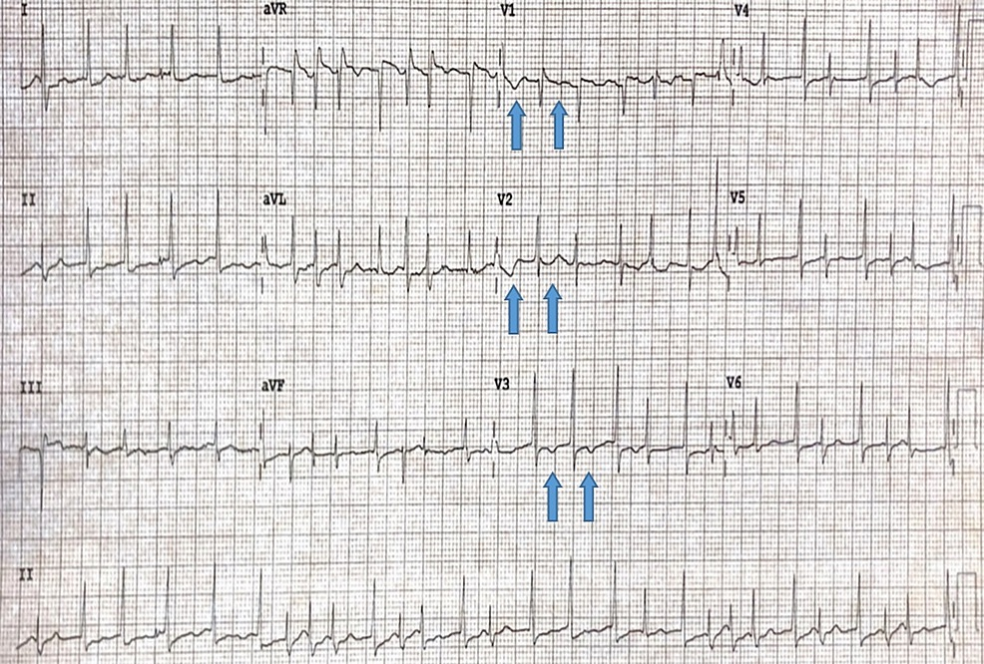
Electrocardiogram on presentation showing atrial fibrillation with a rapid ventricular response (heart rate at 183 bpm), with repolarization abnormalities including T-wave inversion in the precordial leads (blue arrows).

A 69-year-old female with a past medical history of hypertension, hyperlipidemia, and AF status post successful catheter ablation two years ago, presented with chest tightness, palpitations, and lightheadedness after receiving her second evolocumab (monthly dose of 420 mg/3.5 mL) injection. She reported a similar episode with a heart rate (HR) of 230 beats per minute (bpm) after receiving her first evolocumab injection one month ago due to hyperlipidemia with statin intolerance. She denied any prior episodes of AF since her catheter ablation. She also denied any other triggers including emotional and physical stress, heavy alcohol or caffeine drinking, or recent infections. Electrocardiogram (ECG) showed AF with HR of 183 bpm, and T-wave inversion in the precordial leads (Figure 1). The troponin level was normal. Diltiazem drip was started and converted her heart rhythm to normal sinus rhythm. Patient symptoms subsided and ECG changes resolved. She was subsequently discharged on atorvastatin and ezetimibe after stopping evolocumab. The patient denied any recurrent episodes upon three-months follow-up and reported an average HR of 80 bpm.

## Discussion

Evolocumab is a promising anti-PCSK9 monoclonal antibody that decreases the LDL Cholesterol (LDL-C) levels by promoting degradation of PCSK9 enzyme resulting in upregulation and recycling of LDL receptors with subsequent removal of plasma LDL-C by endocytosis. It has been recently approved by FDA after several clinical trials (OSLER, FOURIER) that showed favorable cardiovascular outcomes with a consistent safety profile [2]. Common reported adverse reactions include nasopharyngitis, URTI, and diabetes.

Drug-induced AF is more likely to occur with advanced age, alcohol use, thyroid dysfunction, heart disease, and sleep apnea. It has been associated with cardiovascular drugs, anti-neoplastic agents, antimicrobials, and corticosteroids. Though it is likely to be a very uncommon side effect, AF was reported in the Global Assessment of Plaque Regression with a PCSK9 Antibody as Measured by Intravascular Ultrasound (GLAGOV) trial and its extension trial in 6/484 (1.24%) and 5/770 (0.65%) of the patients receiving evolocumab, respectively [3]. However, the pooled safety analysis from the evolocumab Program to Reduce LDL-C and Cardiovascular Outcomes Following Inhibition of PCSK9 In Different Populations (PROFICIO) program shows a positive benefit-risk profile for evolocumab with similar overall adverse events rates between evolocumab and control groups. It also demonstrates that evolocumab therapy was not associated with significant risk for arrhythmias, hepatotoxicity, muscle-related adverse events, or neurocognitive events. Although the risk seems non-significant and negligible, it can be life-threatening. In our report, the patient suffered from AF with rapid ventricular response upon receiving evolocumab injection twice and presented with hemodynamic instability that resolved after stopping the drug. Unfortunately, the mechanism of this possible adverse reaction is not quite clear. However, our proposed explanation for this possible association in a patient with a left atrial catheter ablation two years prior to presentation is based on recent studies that showed intense expression of PCSK9 in the zone bordering the ischemic or infarct areas in mice hearts, determining the development of infarct size, cardiac function, and autophagy. 

This case highlights the significance of post-marketing surveillance, and the importance of considering the possibility that a patient’s arrhythmia could be drug-induced. More research is needed to clearly explain the mechanism of this possible drug-induced adverse reaction.

## Conclusions

Evolocumab is a novel PCSK-9i that has been recently been FDA-approved as an LDL-lowering agent. Studies have shown its favorable cardiovascular benefits as it lowers the risk of myocardial infarction, stroke, and coronary revascularization in individuals with established atherosclerotic cardiovascular disease. However, it can be associated with rare side effects. Our aim is to increase awareness of such side effects and to highlight the importance of post-marketing surveillance.
